# Some problems with particularism

**DOI:** 10.1007/s11229-022-03948-9

**Published:** 2022-10-29

**Authors:** Keith Raymond Harris

**Affiliations:** Ruhr-Universität Bochum, Bochum, Germany

**Keywords:** Applied epistemology, Consensus, Conspiracy theories, Epistemic authority, Pessimistic induction

## Abstract

Particularists maintain that conspiracy theories are to be assessed individually, while generalists hold that conspiracy theories may be assessed as a class. This paper seeks to clarify the nature and importance of the debate between particularism and generalism, while offering an argument for a version of generalism. I begin by considering three approaches to the definition of conspiracy theory, and offer reason to prefer an approach that defines conspiracy theories in opposition to the claims of epistemic authorities. I argue that particularists rely on an untenably broad definition of conspiracy theory. Then, I argue that particularism and its counterpart are best understood as constellations of theses, rather than a pair of incompatible theses. While some particularist theses are highly plausible, I argue that one important particularist thesis is false. The argument for this conclusion draws on the history of false conspiracy theories. I then defend this conclusion against a pair of potential objections.

## Introduction

The philosophy of conspiracy theories is largely organized around a distinction between *generalism* and *particularism*. Roughly, particularists maintain that conspiracy theories are to be assessed individually. Generalism, in contrast, allows that conspiracy theories may be assessed as a class. Relative to other organizing debates in philosophy—internalism versus externalism in epistemology, for instance—the contest between generalism and particularism is, at least superficially, more lopsided. Particularists often suggest that particularism enjoys something approaching consensus in philosophy (Basham, 2018a; Dentith, 2019, p. 2244; Pigden, 2018). Moreover, there are few if any explicit defenses of generalism in the literature.^1^ It might be tempting to conclude that, whatever debate once existed between particularists and generalists, the debate has now been all but settled. This conclusion would be premature.

The debate between generalism and particularism is less straightforward than the preceding remarks might suggest. First, particularism is often defended by appeal to an untenably general definition of conspiracy theory. Second, a range of theses have been associated with particularism. Some of these are plausible, but others are subject to serious objection. Finally, in contrast to the claims of particularists, the poor historical track record of conspiracy theories warrants a defeasible skeptical attitude toward such theories as a class. Beginning in Sect. 2, I establish these points in turn. Much of the existing philosophical work on conspiracy theories centers on the definition of the term, and so I begin with a substantial discussion of this issue.

To conclude this introduction, it is worth noting that the apparent popularity of particularism in philosophy may be misleading. Assuming that a generalist skepticism toward conspiracy theories is the default position among philosophers, the dearth of material defending generalism may simply reflect the attitude that generalism needs no defending. From this perspective, the apparent popularity of particularism may actually reflect the view that generalism is commonsensical. One who endorses this perspective may question the value of arguing against particularism. But disengagement with particularism on the part of generalists would be a mistake. First, as I argue below, some particularist theses are highly plausible. Second, and relatedly, a failure to indicate where particularism goes wrong may allow for relatively strong particularist theses to take on an unwarranted air of plausibility. Finally, as philosophers have often stressed, the knowledge belonging to a domain is not simply a matter of the mental states of individuals but may be partially realized by material stores of information (Bird, 2010, 2014; Harris, 2021; Popper, 1972). If this view is correct, then there is epistemic value in defending, in print, widely held views that have been challenged in print.

## Defining ‘conspiracy theory’

Much of the existing philosophical work on conspiracy theories aims to define the term *conspiracy theory*. My purpose in this section is not to decisively argue for a particular definition of conspiracy theory, but instead to define a class of theories that could reasonably be called the class of conspiracy theories, and at least stands within or substantially overlaps the class of all conspiracy theories.

### A broad definition

A natural starting point is the broad definition, favored by many particularists, according to which conspiracy theories are theories that allege conspiracies—where conspiracies are typically understood as morally suspect secret plans by groups to influence events (Pigden, 2006, p. 157). Given this definition, it is understandable that particularists think conspiracy theories are not especially problematic. Many widely accepted explanations cite morally dubious secret plans, and it is plausible that explanations of this type can only be assessed according to their individual merits, rather than as a class.

The principal difficulty with the broad definition favored by particularists is that, as even some particularists acknowledge (Coady, 2006, pp. 16–117) it fails to distinguish between conspiracy theories and non-conspiracy theories in a way that aligns, even approximately, with ordinary usage of the term (Cassam, 2019, Chap. 1). For example, the official explanation according to which the events of September 11 were plotted and carried out by individuals working on behalf of Al Qaeda, despite invoking a conspiracy, is not typically regarded as a conspiracy theory. Likewise, many historical explanations that invoke secret and morally dubious plans are not typically regarded as conspiracy theories. The broad definition of conspiracy theory favored by particularists thus appears untenable.

Given that terms can be misused, one might wonder why ordinary usage should carry authority concerning definitions of conspiracy theories. However, this concern rests on a mistaken conception of the significance of appeal to ordinary usage. For most purposes, including present ones, we are primarily concerned with the status of the individual theories regarded as conspiracy theories, and not the class to which they belong. The term *conspiracy theory* is useful only insofar as it identifies those theories. We thus ought to, on pain of unproductively changing the subject, maintain to the extent possible conformity with ordinary usage of the term conspiracy theory. This desideratum can only take us so far, however, as it must be allowed that this term, like any other, can be *mis*used. Realistically, then, any definition of conspiracy theory must conform to some uses, and not to others. Many possible definitions meet this criterion. To arrive at a single definition, we require some additional principle for adjudicating among possible definitions. We might, for instance, select that definition which, of all possible definitions, maximizes conformity with ordinary usage.^2^ One shortcoming with this strategy is that it is simply unclear which definition satisfies this condition.^3^ In what follows, I take it to be a desideratum for any definition of conspiracy theory that it makes the identification of conspiracy theories largely uncontroversial. While it may be controversial whether some conspiracy theory is true, it should not generally be a matter of controversy whether a particular theory is a conspiracy theory. This is a pragmatic desideratum. Unless it is satisfied, the study of conspiracy theories could hardly get off the ground. After all, one could not study the causes, effects, and warrant of conspiracy theories without first being able to identify what theories belong to this class.

### Pejorative definitions

As the example of September 11 conspiracy theories illustrates, a definition of conspiracy theory that largely conforms to ordinary usage must be narrower than the literal definition described above. Two alternatives suggest themselves. The first is a pejorative definition. Such a definition would build in the claim that conspiracy theories are false, lacking in evidence, or something of the like. This proposal seems to better distinguish between theories that are and are not typically regarded as conspiracy theories. Moreover, some empirical evidence suggests that ordinary usage of the term reflects a pejorative definition (Napolitano & Reuter, 2021). However, the proposal faces challenges.

Let us first consider Napolitano and Reuter’s (2021) positive case for a pejorative definition. The authors present a series of empirical studies intended to support, by appeal to patterns of ordinary use and understanding, a pejorative definition. As the authors acknowledge, these patterns do not uniformly support a pejorative definition, as around one third of participants appeared to favor a primarily descriptive definition (2021, p. §4). Beyond this caveat, features of the empirical studies prevent the authors’ results from straightforwardly supporting a pejorative definition. In one study, participants were presented with a vignette in which a character describes a theory as either a conspiracy theory or as a scientific theory and were asked to estimate that character’s attitude toward the theory. Participants assessed the character’s attitude to be considerably more negative when that character described the theory as a conspiracy theory. The authors interpret these results to support the conclusion that the folk understanding of conspiracy theory includes a negative evaluation. However, the results might instead indicate that participants expect attitudes toward conspiracy theories to be more negative than attitudes toward scientific theories.

A similar issue complicates the authors’ interpretation of a corpus analysis of language surrounding ‘conspiracy theory’ on *Reddit* posts. The authors’ found that ‘conspiracy theory’ was consistently attached to negative adjectives, while the neutral term ‘theory’ was associated with far more neutral adjectives. While this study might be taken to support a pejorative definition, an alternative interpretation is that individuals typically have negative attitudes toward conspiracy theories and hence describe such theories in negative terms. Indeed, one might think that if individuals took negative evaluations to be internal to the meaning of conspiracy theory, use of negative adjectives would be redundant.

The authors’ final relevant study presents the most compelling evidence favoring a pejorative definition. In this study, participants were asked to indicate three necessary features of conspiracy theories. The authors found that many participants’ responses included negative features, with around one third of participants including strong negative epistemic features. Still, several aspects of the study preclude it from straightforwardly supporting a pejorative definition. First, while participants tended to include negative features, the brand of negativity—epistemic or otherwise—varied considerably. Second, the study was small—featuring only 50 participants. Finally, it is not obvious that participants would have understood the instruction to report necessary conditions of conspiracy theories. As I have suggested in my remarks on the previous study, it is not easy to tease apart what individuals think conspiracy theories *are* and what individuals think such theories *are generally like*. It is possible that participants tended to report features that they took many or indeed all conspiracy theories to have contingently. In sum, Napolitano and Reuter (2021) present some evidence in favor of a pejorative definition, but this evidence is not decisive.

I have thus far sought to undercut the positive case for a pejorative definition. I now present some problems for pejorative definitions. Consider the suggestion that conspiracy theories are, by definition, false. Perhaps the most obvious concern for this proposal is that some conspiracy theories are true. One such example is the allegation of the existence of an extensive program conducted by the NSA to collect data on American citizens. Prior to the revelation of such a program, such an allegation was regarded as a conspiracy theory. The present proposal, like the broad definition, seems not to accord with ordinary usage of the term. The present proposal might, however, be defended by contending that early descriptions of the above allegation as a conspiracy theory were mistaken, but consistent with the definition insofar as those descriptions were premised on the *belief* that the theory in question was false.^4^ In my view, one difficulty with this defense is that it seems we may still, in retrospect, felicitously say that this was once a conspiracy theory. For those unmoved by this point, the present proposal faces a further difficulty.

On the present proposal, to determine whether a given theory is a conspiracy theory would first require determining its truth value. The resultant difficulty is hardly a decisive theoretical reason to reject the present definition—it might after all be the case that some correct definitions render certain academic pursuits difficult. However, this difficulty does furnish some reason to focus on those theories captured by an alternative definition, whether or not this definition is ultimately taken to be suited to defining conspiracy theory.

Let us turn to an alternative proposal according to which conspiracy theories lack evidence by definition. A first concern for this proposal is related to the concern raised above for defining conspiracy theories as false. Admittedly, it does not follow from the fact that some proposition is true that there is evidence for that proposition. There are, for example, true propositions capturing states of affairs in remote corners of the universe, for which no one has evidence. However, in the case of conspiracy theories, there is reason to think that the existence of true conspiracy theories implies the existence of evidence for these theories. At a minimum, the conspirators—barring unusual circumstances involving memory loss and the like—will have evidence of conspiracy (Harris, 2018). Thus, there is reason to deny that conspiracy theories are lacking in evidence by definition, at least according to one construal of what it is for a theory to be lacking in evidence.^5^

A second concern for this proposal arises if we acknowledge the possibility of misleading evidence. Suppose that there is a false theory that alleges conspiratorial activity. It seems implausible that whether this theory amounts to a conspiracy theory can depend on whether there exists misleading evidence for the theory. One last concern for the present proposal is that it would render certain debates all but senseless from the outset. Consider the ongoing debate as to whether it is ever rational to believe in conspiracy theories. Assuming that rational belief requires evidence, the present definition would quickly settle this debate in the negative. If there is a sensible question to ask as to the rationality of belief in conspiracy theories, the present definition appears incorrect or at least impractical insofar as it would require apparently sensible questions to be reformulated in other terms.

These objections leave open that there might be an alternative definition according to which conspiracy theories are defined in relation to an absence of evidence. One might argue that conspiracy theories by definition are not supported by substantial publicly accessible evidence, for example. I will not attempt here to rule out all definitions of this type. Instead, I turn to an alternative approach that, at a minimum, will allow us to distinguish an important body of theories within or adjacent to the class of conspiracy theories.

### Counter-authority definitions

I have argued that existing evidence does not strongly favor a pejorative definition of conspiracy theory, and I have offered independent reasons to reject pejorative definitions. Let us therefore turn to an alternative approach. Many authors have proposed that conspiracy theories are, by definition, contrary to an official account, where the official account is the one favored by authorities (Coady, 2006, 2007; Feldman, 2011; Harris, 2018; Ichino & Räikkä, 2020; Keeley, 2006; Levy, 2007; Räikkä, 2009). This approach applies most naturally when we consider conspiracy theories that purport to explain historical events. For many historical events, there are competing explanations available. One such explanation is the official account and alternatives are, on this approach, conspiracy theories. Consider again September 11 conspiracy theories. These theories conflict with the official account according to which the events of September 11 were carried out by agents acting on behalf of Al Qaeda. More generally, many conspiracy theories are proposed explanations of events that conflict with competing official accounts of those same events. However, there is reason to think that defining conspiracy theories in relation to official accounts of historical events is too restrictive. First, conspiracy theories sometimes allege plots to influence future events. Conspiracy theorists have long speculated about the intent to establish a New World Order and, in recent years, some conspiracy theorists speculated about plans for a federal takeover of Texas under the guise of the Jade Helm 15 military training exercises (Fernandez, 2015). Such examples pose two problems for the identification of conspiracy theories with explanations of past events inconsistent with official accounts. Most straightforwardly, some conspiracy theories are not proposed explanations of past events. Secondly, as in the New World Order case, there is a conspiracy theory even though there is no clear official account with which that theory conflicts.

With these points in mind, let us consider the proposal that conspiracy theories are theories that allege conspiracies and that conflict with the claims of authorities. Conflict between a theory and the claims of authorities may be explicit, as when authorities directly reject contrarian explanations of the events of September 11. In other cases, conflict may be implicit. Some allegations of conspiracy never receive the direct attention of authorities, but are inconsistent with claims otherwise made by authorities.

There remains ambiguity in the present proposal concerning how authority is to be understood.^6^ On one construal, authority amounts to power. So understood, paradigm cases of authorities are political figures occupying positions of influence. Alternatively, one might construe authority in epistemic terms. So understood, the authority at issue is the kind invoked when an individual is described as an authority on a subject. Given these distinct kinds of authority, we are left with a question concerning the variety of authority in relation to which conspiracy theories are to be defined. While I will not attempt to decisively rule out the construal of conspiracy theories as contrary to the claims of individuals in power, I offer some reason to favor the alternative definition or to at least recognize the existence of an important class of conspiracy theories that conflict with the claims of epistemic authorities.

One reason to deny that conspiracy theories must by definition conflict with the claims of authorities—where authority is construed as power—is that such authorities can and do engage in conspiracy theorizing. For example, the claim that Joe Biden won the 2020 US presidential election only due to widespread electoral fraud is widely regarded as a conspiracy theory.^7^ However, this theory was repeatedly alleged by Donald Trump, both during and after his presidency. We thus have a clear example illustrating that persons in power can engage in conspiracy theorizing. This example is not an isolated case. Consider the routine allegations of conspiracy against George Soros made by Central European heads of state (Plenta, 2020). In fact, allegations of conspiracy are common in the discourse of political leaders, especially populists (Kazin, 2017), and many such allegations are regarded as conspiracy theories. In fact, it seems appropriate to say that policy can be more or less driven by conspiracy theories. Conspiracy theories can in principle, and sometimes do, influence the direction of political power. When considered in this light, the definition of conspiracies theories in opposition to the claims of those in power appears misguided.

This brings us to the definition of conspiracy theories as contrary to the claims of relevant epistemic authorities (cf. Levy, 2007). Some authors understand epistemic authority as consisting in, or deriving from, reliability (Brown, 2009; Goldman, 1999). On this construal of epistemic authority, the definition of conspiracy theories as conflicting with the claims of epistemic authorities would amount to a pejorative definition, and would inherit the weaknesses of such definitions. There are thus reasons to avoid defining conspiracy theories as conflicting with the claims of epistemic authorities, so construed.

I will instead understand epistemic authority in terms of credentials, positions, and the like, rather than in terms of reliability. On this approach, the reliability of epistemic authorities will be contingent upon whether credentials and positions are reserved for those who are epistemically reliable. In well-functioning systems, only those who will be reliable judges about claims in a certain domain will receive credentials or positions constitutive of epistemic authority in that domain. For example, in a well-functioning system, only individuals who are reliable judges of questions concerning biochemistry will receive advanced degrees in biochemistry. Likewise, in a well-functioning system, only individuals with a high degree of reliability concerning questions related to engineering will occupy prominent roles within engineering associations. This is not to say that reliability concerning a subject matter should always precede epistemic authority with respect to that subject matter. For example, it may be the occupation of a certain role that allows an intelligence analyst to be a reliable judge about a certain subject matter—because that role provides the analyst with access to otherwise inaccessible evidence. However, in a well-functioning system, only those who will be reliable judges when occupying positions of epistemic authority hold those positions. In a poorly functioning system, by contrast, individuals may occupy positions of epistemic authority by virtue of nepotism, bribery, or some further factor unrelated to reliability.

Having clarified the concept of epistemic authority, we may define conspiracy theories as theories that allege conspiracies and conflict with the claims of relevant epistemic authorities, where epistemic authority is a matter of credentials and positions. It might be objected that, just as political authorities sometimes engage in conspiracy theorizing, so too do epistemic authorities. Consider the notorious saga of Andrew Wakefield, whose 1998 *Lancet* paper suggesting a link between the MMR vaccine and autism is widely cited among members of anti-vaccination communities (Deer, 2020). This objection highlights the potential ambiguity arising from the bare plural “relevant epistemic authorities” in the present definition. To reduce this ambiguity, and to forestall the present objection, the “claims of relevant epistemic authorities” ought to be understood as the claims of those representing the consensus position among relevant epistemic authorities. To specify precisely when such a consensus exists would require grappling with issues of vagueness that I cannot address here. For the present, it suffices to note three points. First, despite issues of vagueness, clear cases of consensus among relevant epistemic authorities are common. For example, there is a clear consensus among intelligence professionals, journalists, and relevant scientific professionals that the World Trade Center was brought down by planes hijacked by individuals working on behalf of Al Qaeda and there is a clear consensus among relevant epistemic authorities that the Earth is round. Second, such consensuses can exist in the face of dissent from a small number of individuals possessing relevant credentials and positions (Dellsén, 2021), especially where relevant authoritative institutions and organizations reject that dissent. For example, the “Scholars for 9/11 Truth”—a small organization of academics who support the controlled demolition conspiracy theory concerning September 11—has been roundly criticized by other relevant epistemic authorities and has enjoyed little institutional support (Gravois, 2006). Similarly, while Wakefield’s paper suggesting a link between vaccination and autism originally was originally published in a reputable scientific journal, the paper has since been retracted and Wakefield himself is widely condemned within the medical community. Third, where there is no clear consensus inconsistent with an allegation of conspiracy, that allegation does not amount to a conspiracy theory. Thus, where allegations of conspiracy remain highly contested among relevant epistemic authorities, those allegations that are substantially supported by some such authorities do not amount to conspiracy theories on the present approach.

The proposed definition accounts for why some, but only some, proposed explanations of the events of September 11 are conspiracy theories. Moreover, the present definition captures the common feature of conspiracy theories in various domains. We may regard allegations of widespread electoral fraud in the 2020 US presidential election as conspiracy theories because these conflict with the consensus of election security experts, investigative journalists, and other relevant epistemic authorities. Likewise, we may regard the flat Earth theory as a conspiracy theory because this theory alleges a coverup of the shape of the Earth and conflicts with the claims of relevant scientific authorities. Additionally, the present definition allows for conspiracy theories to be identified with relatively^8^ little controversy, as compared with pejorative definitions. This is because even those that deny the reliability of those holding certain positions and credentials may nonetheless recognize them as epistemic authorities, in the relevant sense.

The proposed definition of conspiracy theory, like similar definitions (Harris, 2018), implies that the same theory may or may not count as a conspiracy theory depending on when and where it is considered. The allegation that the NSA has engaged in large-scale spying on American citizens may have been a conspiracy theory, but no longer. The allegation that Russian hackers penetrated the US power grid via a Vermont-based utility was not a conspiracy theory when first brought to public attention. However, given changing attitudes among relevant epistemic authorities, it is now a conspiracy theory. In short, the proposed definition effectively relativizes the designation as conspiracy theory.

One might prefer an alternative definition of conspiracy theory. I will not attempt to offer a decisive argument against alternative definitions here—I doubt that a decisive argument for any definition can be given. However, even those who prefer an alternative definition of conspiracy theory can acknowledge that an important class of theories allege conspiracies and conflict with the claims of relevant epistemic authorities. For those philosophers who favor a broad definition of conspiracy theory, this class will be a proper subclass of the class of all conspiracy theories. In what follows, I will understand conspiracy theories according to the definition developed in this section and some of the arguments to follow will depend on understanding conspiracy theories thusly. Those who prefer alternative definitions may regard these arguments not as concerning all conspiracy theories, but as concerning either a subclass of conspiracy theories or a class of theories closely related to conspiracy theories. Even for those who prefer an alternative definition, the argument to come—according to which there are general grounds for skepticism of conspiracy theories—will establish that there is reason to be skeptical of individual theories captured by this definition.

## What is particularism?

In Sect. 5, I take up an explicit defense of generalism. However, before making the case for generalism, it is necessary to consider a second concern for particularism. This is a concern, and not an outright objection, for it consists in a lack of clarity as to what particularism claims. While some theses advanced under the banner of particularism border on commonsensical, others are implausible.

Consider first how particularism and generalism were defined upon introduction into the philosophical literature by Joel Buenting and Jason Taylor:

*Generalism*

[T]he rationality of conspiracy theories can be assessed without considering particular conspiracy theories. (2010, p. 568)

*Particularism*

[D]enies that the rationality of conspiracy theories can be assessed without considering particular conspiracy theories. (2010, pp. 568–569)

Elsewhere, the authors make clear that “considering particular conspiracy theories” amounts to considering the evidence bearing on particular conspiracy theories (2010, p. 570).

Buenting and Taylor’s presentation of generalism and particularism suggests that these theses concern the assessment of theories, as opposed to the assessment of beliefs in conspiracy theories. An analogy may help to clarify this distinction. Philosophers and theologians often debate the case for belief in God. These debates center on the relative strengths of certain arguments, including design and cosmological arguments, and the argument from evil. But, in addition to evaluations of such arguments, there are separate questions to be asked concerning the rationality of individuals' beliefs concerning the existence of God. Whether individual beliefs in God are rational may depend on whether and how such beliefs are influenced by the aforementioned arguments, but also may also depend on how they are influenced by sociological and psychological factors including propensities toward wishful thinking, respect for or hostility toward religious authorities, and so on. Thus, one might for example find the case for belief in the existence of God compelling, even while deeming most religious beliefs irrational.

As in the religious case, we can distinguish between assessments of conspiracy theories and assessments of beliefs in conspiracy theories. Buenting and Taylor present themselves as concerned with the former. However, this presentation is not consistent among defenders of particularism. For instance, Dentith describes the state of philosophical literature on conspiracy theories as follows:

[T]he current findings in the Philosophy of Conspiracy Theories (to coin a new discipline) simply show that *belief* in conspiracy theories is not prima facie irrational. (2016, p. 2, *emphasis added*)

Moreover, while there are few if any avowed generalists, large swaths of academic work take a critical stance toward belief in conspiracy theories. This work principally takes the form of psychological research (Abalakina-Paap et al., 1999; Brotherton & French, 2014; Cichocka et al., 2016; Darwin et al., 2011; Douglas et al., 2016; Swami et al., 2011; Van Prooijen, 2018; Van Prooijen et al., 2018) and explorations of the connections between epistemic vice and belief in conspiracy theories (Cassam, 2016; Harris, 2018; Meyer, 2019). That such work is routinely challenged by particularists (Basham & Dentith, 2018; Basham, 2018b; Hagen, 2018, 2020) suggests that particularists are concerned not only to defend conspiracy theories, but to defend belief in conspiracy theories.

Given the distinct projects taken up by particularists, how are we to understand particularism and generalism? A natural approach is to understand particularism as comprising a constellation of theses. Particularism, as defined by Buenting and Taylor, asserts that conspiracy theories are to be assessed by considering the evidence bearing on particular *theories*. We may recognize another particularist thesis according to which there are no general reasons to doubt the rationality of *beliefs* in conspiracy theories. For each particularist thesis, we may define a corresponding and conflicting generalism.

Approached in this way, particularism and generalism are not simply a pair of mutually inconsistent theses. The particularism versus generalism debate is better understood by analogy with other organizing debates familiar to philosophers. In epistemology, for example, the debate over externalism and internalism is not a conflict between two mutually inconsistent theses. Externalism and internalism are associated with various theses such that one may be an externalist in one respect and an internalist in another. Similarly, one might in principle think that conspiracy theories can only be adequately assessed by considering the evidence bearing on each *theory*, while also maintaining that there is strong reason to doubt the rationality of any particular *belief* in a conspiracy theory, insofar as such beliefs are typically caused by epistemic vice, cognitive bias, or something of the sort.^9^

It might be objected that I have overstated the separability of what I have described as distinct particularist and generalist theses. After all, the proper means of assessing conspiracy theories no doubt bears on whether beliefs in conspiracy theories are typically irrational. If there are general reasons to doubt conspiracy theories, then beliefs in conspiracy theories will typically be irrational, or so one might think. However, this line of objection rests on an oversimplification. It may well be that many of those who take a critical attitude toward beliefs in conspiracy theories—for instance those who propose to account for such beliefs in terms of epistemic vice or cognitive bias—do so in part because of a skeptical attitude toward conspiracy theories themselves. But what matters for the rationality of a subject’s belief in a given conspiracy theory is not whether there *is* reason to doubt or affirm it. Rather, what matters is whether the subject *has* reason to doubt or affirm it.

Before concluding this section, it is worth acknowledging an element of the motivation for particularism that has not been evident so far. Many particularists worry that a dismissive attitude toward conspiracy theories is dangerous, insofar as such an attitude would allow for conspiracies to remain hidden. Such a concern is evident in the following passage:

Those who are excessively unwilling to believe in conspiracy harm us all by making it easier for conspirators to remain undetected. (Coady, 2007, p. 196)

Pigden (2006, pp. 165–166) and Dentith (2016) express similar concerns. It should be noted that these authors are operating with a relatively broad definition of conspiracy theory, and so are concerned that a reflexive dismissal of allegations of conspiracy is dangerous. What I want to draw attention to here is that particularists are partly motivated by practical considerations. Relatedly, particularists sometimes endorse a prescription along the following lines:

I advocate the alternative strategy of not dismissing conspiracy theories out of hand, simply because they are conspiracy theories, but of being prepared to investigate them and even to believe them if that is what the evidence indicates. (Pigden, 2007, p. 221)

Pigden’s contention is that conspiracy theories should be investigated, rather than ignored merely because they are conspiracy theories. This may be understood as a further particularist thesis insofar as it recommends investigation of the details of particular conspiracy theories. One might define a corresponding generalist position according to which it is not the case that one should investigate individual conspiracy theories.

As I have emphasized above, various particularist theses may stand or fall independently of one another. This practical thesis is no different—whether one ought to investigate conspiracy theories may depend on pragmatic or moral considerations largely independent of the evidence for and against those theories.^10^ The distinct particularist theses are not always clearly distinguished in existing scholarship, a fact that has likely contributed to misunderstandings among commentators. In what follows, I will be principally concerned with the particularist theses according to which conspiracy theories are to be assessed solely according to the evidence that bears upon them. I will in what follows use particularism to refer to this thesis, unless otherwise indicated. In the next section, I distinguish between versions of this thesis of varying strengths.

## Weak and strong particularism

Some particularist theses are highly plausible. Consider, for instance, the following passage from Buenting and Taylor:

Judging any theory to be insufficient independently of considerations regarding the evidence is irrational. Thus, a rejection of conspiracy theories simpliciter seems irrational; rational rejection or acceptance of a theory must supervene on the quality of evidence for or against that theory. (2010, p. 570)

Elements of this passage are misguided for reasons familiar from the discussion in Sect. 3. What determines whether rejection or acceptance of a theory is rational is not strictly the evidence for or against that theory, but what evidence the subject possesses. Setting this quibble aside, Buenting and Taylor present a plausible version of particularism according to which conspiracy theories are to be assessed according to the evidence bearing upon them. This, as the authors point out, is a straightforward consequence of the more general claim that the adequacy of theories in general depends on the evidence. The version of particularism that follows from this weak form of evidentialism is, to my mind, unobjectionable. Moreover, to my knowledge, no philosopher has disputed this thesis.

However, even when we focus purely on how conspiracy theories are to be assessed, particularists do not confine themselves to this weak claim. Dentith, for example, writes that:

Philosophers interested in the topic of belief in conspiracy theories (with few exceptions) have argued that you cannot principally assess conspiracy theories as a class but, rather, we must undertake such an analysis on a case-by-case basis. The *prima facie* suspicion of conspiracy theories *generally* before assessing the *particulars* of individual theories, gets things back to front. (Dentith, 2019, p. 2244)

Whereas Buenting and Taylor’s claim is that conspiracy theories must be assessed according to the evidence, Dentith contends that a general suspicion of conspiracy theories as a class is misguided. With this in mind, we may distinguish between the following two theses:

*Weak Particularism*

The proper assessment of any given conspiracy theory supervenes on the evidence for and against that theory.

*Strong Particularism*

The proper assessment of any given conspiracy theory supervenes on the evidence for and against that theory *and* there is no evidence against the truth of conspiracy theories as a class.

It is consistent with *Weak Particularism*, but not *Strong Particularism*, that there is general evidence against conspiracy theories. If there is such evidence then, although conspiracy theories can only by assessed by considering the evidence bearing upon them, there is reason for a general suspicion of conspiracy theories.

Beginning in Sect. 5, I will argue that *Strong Particularism* is false—there is evidence against conspiracy theories as a class. Before doing so, it is worth making two points. First, as I have emphasized above, the definition of conspiracy theories I have adopted here is narrower than the one typically adopted by proponents of particularism. Thus, particularists may attempt to sidestep the conclusion that strong particularism is false by claiming that it tells us nothing about conspiracy theories, as particularists propose to understand them. This would be a mistake, in my view, because the definition of conspiracy theory adopted here largely conforms with common usage and because the present definition at least picks out an important class of theories within or overlapping with the class of all conspiracy theories. Second, *Strong Particularism* is central to the project undertaken by particularist philosophers. To see this, consider the following passage:

The idea that conspiracy theories as such are somehow intellectually suspect is a superstitious or irrational belief, since there is no reason whatsoever to think it true. It is an idiotic superstition since a modicum of critical reflection reveals that it is false. (Pigden, 2006, p. 165)

Pigden, like Dentith, is committed to the non-existence of general grounds for suspicion of conspiracy theories. Indeed, Pigden suggests that the non-existence of such grounds is obvious. More generally, particularists are critical of the tendency to treat the fact that some theory is a conspiracy theory as a reason to doubt it (Dentith, 2019; Pigden, 2007). Such criticism may be in order when one understands conspiracy theories merely as theories alleging conspiracies. However, as I now argue, there is good reason for generalized skepticism of conspiracy theories, as understood here. Thus, *Strong Particularism* is false. While the first argument targets *Strong Particularism*, the second argument to follow provides some reason for caution about application of *Weak Particularism*.

## Two pessimistic meta-inductions for conspiracy theories

The case for generalized skepticism concerning conspiracy theories resembles the pessimistic meta-induction in philosophy of science. In short, the argument appeals to the history of false conspiracy theories to warrant skepticism of other conspiracy theories. This appeal to the history of false conspiracy theories mirrors previous defenses of particularism, many of which appeal to history. Consider the following example:

Our estimates as to how independently likely conspiracies are varies over time. Certainly, post the revelations of the NSA’s mass surveillance programme by Edward Snowden in 2013 claims of large-scale, political conspiracy have been treated much more sympathetically, and considered more likely by ordinary reasoners; it appears people underestimated how independently likely it was that a major, political conspiracy was happening here and now.

Working out the true prior probability or independent likeliness of claims of conspiracy being in amongst the pool of credible explanatory hypotheses will be, of course, difficult. However, it is fair to say that people either underestimate or underplay both historical and contemporary accounts of events which cite conspiracies as salient causes. (Dentith, 2016, pp. 12–13)

Part of the excerpted passage might be read as defending the purely descriptive thesis that estimates of the prior probability of any given conspiracy theory being true have tended to vary as real-world conspiracies have been revealed. However, Dentith’s suggestion that people have tended to underestimate the prior probability of conspiracy makes clear that Dentith takes history to provide reason to assign a higher prior probability^11^ to conspiracy theories than individuals might otherwise be inclined to do.^12^

Assessing Dentith’s appeal to history is complicated by the broad definition of conspiracy theory Dentith favors. In fact, prior instances of conspiracy plausibly do offer reason to assign relatively high prior probabilities to further allegations of conspiracy, insofar as they offer evidence of the human tendency to conspire. However, the existence of a conspiracy does not guarantee the existence of a true conspiracy theory, as defined here. Hence, while the appeal to history may support the assignment of relatively high prior probabilities to conspiracy theories given a broad definition, it is not clear that it does so given the definition adopted here.

Given the definition of conspiracy theory adopted here, history will recommend the assignment of relatively high prior probabilities to such theories, if at all, partially because of historical instances of true conspiracy theories. However, even a large number of true conspiracy theories would not be enough to warrant assignment of relatively high prior probabilities to further conspiracy theories. What is needed to warrant such assignment is the premise that the proportion of conspiracy theories that are true meets some threshold. Herein lies the greatest challenge to the argument now under consideration. When we consider the history of conspiracy theories, we are met with an overwhelmingly high proportion of false conspiracy theories. History warrants a strong presumption of falsity for further conspiracy theories.

Some readers will accept, already, that the vast majority of past conspiracy theories have been false. Such readers thereby already accept the key premise of the pessimistic inductions. But we may also motivate the claim that past conspiracy theories have overwhelmingly been false by mustering empirical and theoretical considerations. The first point to reflect upon here is the sheer number of conspiracy theories. Even the most rigorous empirical study could not yield a precise count of the number of extant conspiracy theories. This is partly because it is not always clear how to individuate conspiracy theories and in part because the existence conditions for theories are not entirely clear. It is not obvious, for example, whether it is enough for a conspiracy theory to exist that some individual considers an allegation of conspiracy that conflicts with the claims of epistemic authorities, whether some individual believes such an allegation, or, indeed, whether the existence of conspiracy theories depends in any way on the attitudes of individuals. Still, even using relatively a relatively stringent criterion for the existence of conspiracy theories—say the requirement that multiple individuals sincerely profess belief in the allegation of conspiracy—conspiracy theories abound. Moreover, there are general reasons to think that conspiracy theories are even more common than is sometimes supposed.

Recent work in the psychology of conspiracy theories might lead one to underestimate the volume of conspiracy theories. Studies have indicated, for instance, that individuals are more likely to find allegations of conspiracy plausible where these allegations surround momentous events (Leman & Cinnirella, 2007). One might therefore conclude that most conspiracy theories concern momentous events and ultimately that, because such events are rare, the total number of conspiracy theories is low. Admittedly, the most notorious conspiracy theories often surround momentous events—here we need only think of September 11 conspiracy theories, conspiracy theories denying the reality of the Holocaust, and conspiracy theories surrounding the COVID-19 pandemic. But it would be a mistake to suppose that most conspiracy theories concern momentous events. There are vastly more non-momentous events than momentous events and thus, even if each momentous event is more likely to result in conspiracy theories than a corresponding non-momentous event, most conspiracy theories might nonetheless concern non-momentous events. Conspiracy theories concerning momentous events likely come to mind more readily, but focusing on such events threatens to underappreciate just how many events give rise to conspiracy theories. Consider some recent examples. In late March of 2021, the “Ever Given” cargo ship ran aground, blocking the Suez Canal for several days. During this period, several conspiracy theories linking the ship to Hillary Clinton emerged (Petrocelli, 2021). At the end of his presidency, Donald Trump delivered his farewell address from a stage decorated with 17 flags. Because Q is the 17th letter of the alphabet, some adherents of the QAnon conspiracy theory speculated that the flags were a confirmation of elements of the broader QAnon myth (Griffin, 2021). These conspiracy theories have received less attention than pandemic and election conspiracy theories circulating during the same period, but are no less real. More generally, as the case of the 17 flags illustrates, even the least significant events and states of affairs are sometimes explained by appeal to conspiracy theories.

It might be objected here that allegations of conspiracy surrounding non-momentous events will often go unnoticed by epistemic authorities, and hence will not constitute conspiracy theories on the present approach.^13^ However, even if an allegation of conspiracy goes unnoticed by relevant epistemic authorities, it may nonetheless be inconsistent with the claims of those authorities. Consider the case of the Ever Given blocking the Suez Canal. Relevant epistemic authorities did not explicitly reject conspiratorial claims that the incident had something to do with Hillary Clinton. However, the explanation provided by relevant epistemic authorities—that the ship became grounded due to combination of poor weather conditions and piloting errors (Yee & Glanz, 2021)—is inconsistent with such conspiratorial explanations. Indeed, although many mutually inconsistent explanations were suggested by epistemic authorities early on (Jankowicz, 2021), the fact that these explanations were uniformly inconsistent with allegations of conspiracy is enough to identify such allegations as conspiracy theories on the present approach.

That conspiracy theories often arise to explain entirely insignificant events and states of affairs is just one reason to think such theories are far more common than might be supposed. It is also worth noting the following points in this connection. First, certain event types reliably give rise to conspiracy theories. One need only consider conspiracy theories surrounding assassinations, mass shootings, and pandemics. Second, certain individuals generate large quantities of conspiracy theories. Notorious examples include Alex Jones and David Icke. Finally, the lives of certain individuals inspire large numbers of conspiracy theories. Adherents of the QAnon conspiracy theory have speculated that Joe Biden was secretly allied with Donald Trump, was replaced with a hologram, was replaced with a clone, or was replaced with the actor James Woods (Palmer, 2021). This only scratches the surface of the enormous body of conspiracy theories to emerge in recent years. Reflecting on the sheer number of conspiracy theories, it is clear that the mere fact that some conspiracy theories have proven true is not sufficient to assign relatively high prior probabilities to conspiracy theories. When one considers that the conspiracy theories discussed thus far in this section make up just a small portion of the vast number of preposterous conspiracy theories, one can conclude that the vast majority of conspiracy theories have been false,^14^ even if a precise proportion cannot be determined.

Just as variations of the pessimistic meta-induction concerning scientific theories have been proposed, one can draw from the track record of conspiracy theories various conclusions for the appropriate attitude toward other conspiracy theories. Most straightforwardly, one might conclude from the fact that the overwhelming majority of conspiracy theories have been false that conspiracy theories now under consideration are overwhelmingly likely to be false, even absent consideration of the evidence specific to those theories. Such a line of argument parallels the pessimistic meta-induction floated by Hilary Putnam (1978), which concludes form the history of false past scientific theories that contemporary scientific theories are likely to be false. This line of argument has been criticized in the scientific context, on the grounds that scientific realism posits the convergence of science on truth, and is hence consistent with the falsity of most past theories (Lewis, 2001, pp. 272–273). However, there is little reason to suppose that contemporary conspiracy theories are true at a higher rate than past conspiracy theories. Thus, even if the present line of defense against the pessimistic meta-induction succeeds in the context of science, there is little reason to think it succeeds in the case of conspiracy theories.

Thus, we have an argument against *Strong Particularism* and for what we might regard as a weak form of generalism. In denying *Strong Particularism*, we need not deny that the appropriate way of assessing conspiracy theories is through consideration of the evidence. On the contrary, the argument here shows that the poor track record of conspiracy theories offers grounds for a presumption of doubt toward such theories as a class. Notably, in vindicating this presumption of doubt, we vindicate at least to some degree the dismissive attitude toward conspiracy theories so often criticized by particularists. This is not to say that conspiracy theories can never be rationally believed. Rather, the upshot of the argument here is that there is reason to assign a low probability to individual conspiracy theories prior to considering the evidence bearing specifically on individual theories. Such a conclusion is consistent with the possibility that one can sometimes have sufficient evidence favoring a conspiracy theory to believe that theory rationally.

It might be objected that the pessimistic meta-induction suggested above is uncharitable to conspiracy theories and those who believe them. There are undoubtedly vast numbers of false conspiracy theories, but not all conspiracy theories are equal, and the most preposterous do not discredit all other conspiracy theories. To understand how this objection errs, one must keep in mind that the argument above is intended only to motivate the assignment of relatively low probabilities to conspiracy theories *before the details of those theories are considered*. However, the distinction between preposterous and non-preposterous conspiracy theories will be apparent only when those theories are examined more closely, compared against common sense, compared against available evidence, and so on. At this stage, some theories will perform better than others. However, the distinction at this later stage does not undermine the case for initial assignment of low prior probabilities to conspiracy theories in general.

I have thus far argued that *Strong Particularism* is false. I now argue that implementation of even *Weak Particularism* calls for caution. Consider an alternative version of the pessimistic meta-induction for conspiracy theories. Just as the previous argument was based on a version of the pessimistic meta-induction formulated by Putnam (1978), this latter argument is based on a version formulated by Larry Laudan (1981). Laudan’s argument aims to show not strictly that contemporary scientific theories are likely false, but that the empirical success of contemporary scientific theories is not a reliable indicator of truth. The basis for this argument is the body of past false scientific theories that were nonetheless empirically successful. Given this history, Laudan concludes that empirical success is not a reliable indicator of truth.

Peter J. Lewis (2001) argues that Laudan fails to exclude the possibility that, while empirical success is a reliable indicator of truth, there have simply been so many false scientific theories that many of them have proven empirically successful, at least for a time. Empirically successful false theories would then be like the many false positives that might be output by even a reliable test, provided that the test is applied in an environment populated by many negative cases.

Regardless of whether Laudan’s version of the pessimistic meta-induction succeeds in the context of science, a parallel argument can be applied to conspiracy theories. Whereas Laudan is concerned with the reliability of empirical success as the test for truth in science, the corresponding test in the case of conspiracy theories is conformity to evidence. Conspiracy theories are not generally motivated by the ability to make novel predictions, but rather by the ability to account for data left unexplained by official narratives or the perspectives of epistemic authorities more generally (Keeley, 2006; Harris, 2018). With this in mind, we may ask, in a manner parallel to Laudan’s query concerning the reliability of empirical success as a test for truth, whether conformity to evidence is a reliable test for truth in the case of conspiracy theories. Notably, whereas Laudan’s pessimistic meta-induction may be criticized for lacking the appropriate premise—this being that most false scientific theories have nonetheless been empirically successful (Lewis, 2001)—we have, in the case of conspiracy theories, the needed empirical basis for a second pessimistic meta-induction. It is not only that most conspiracy theories that have conformed to the evidence have been false. It is also the case that most false conspiracy theories have conformed to the evidence.

As many commentators have observed, conspiracy theories are resistant to falsification. This is because conspiracy theories can fold apparently recalcitrant evidence into the conspiracy narrative. An illustrative example is the tendency among proponents of September 11 conspiracy theories to dismiss critics of the conspiracy theory as complicit in a coverup, if not the underlying conspiracy (Levin & McKenzie, 2009). It may be tempting to regard the resistance of conspiracy theories to falsification as itself problematic for such theories. However, as others have noted, falsification may not be an appropriate standard by which to judge theories that allege conspiracies (Basham, 2006; Keeley, 2006). Yet even if resistance to falsification is not itself a mark against conspiracy theories, this resistance is due to the ability of conspiracy theories to conform to apparently recalcitrant evidence. This ability implies that even those conspiracy theories that appear preposterous to outside observers can be made to conform to the available evidence. Often, as in the case alluded to above, achieving this conformity will require reframing the dissent of epistemic authorities as evidence of the expansiveness of the conspiratorial plot.

In virtue of the ability of conspiracy theories to conform to nearly any available evidence—a feature sometimes lauded by particularists (Basham, 2011, p. 63)—most false conspiracy theories nonetheless conform to the evidence. In the present context, this is important for two reasons. First, it follows from the fact that most false conspiracy theories nonetheless conform to the evidence that conformity to evidence is not a reliable test for the truth of conspiracy theories. In other words, we have the required premise for a pessimistic meta-induction styled after Laudan’s. Second, I have noted above that the denial of *Strong Particularism* does not require rejection of the claim that conspiracy theories should be assessed according to the evidence. The present line of argument complicates this picture, however, for it suggests that practically all conspiracy theories might perform well with respect to the evidence, provided that one makes the appropriate background assumptions. The implications of this point are not entirely clear, but it would be premature to conclude that conspiracy theories should not be assessed according to the evidence. It may instead be that we require some constraints on how evidence—especially in the form of testimony from epistemic authorities—can legitimately be interpreted. I do not pursue the issue here.

To conclude this section, let us state plainly what the pessimistic meta-inductions presented here show. Because we have defined conspiracy theories as theories that conflict with the claims of epistemic authorities, we may take our first argument to indicate that believing allegations of conspiracy that conflict with such claims is likely to lead one to error. Thus, insofar as we aim to have true beliefs and to avoid false ones, we have reason for an initial skepticism of conspiracy theories. Our second argument illustrates that the conformity of conspiracy theories with evidence is not a reliable indicator of the truth of such theories. Thus, one should not too readily accept even those conspiracy theories that appear to conform to the evidence.

## Objections and replies

I now consider two remaining concerns about the argument against *Strong Particularism* developed here. Defenders of *Strong Particularism* are likely to allege that this argument is both mistaken and dangerous. The argument is mistaken, one might argue, because epistemic authorities need not be reliable. After all, I have explicitly declined to define epistemic authority in such a way as to require reliability. The argument is dangerous, one might argue, because it seems to encourage blind faith in epistemic authorities, whose overriding motive may be to protect malign interests, rather than to discover and disseminate the truth. Let us consider both objections in turn.

Whether or not epistemic authorities are reliable is an empirical question. Moreover, it is a question for which there is no single answer. Epistemic authorities may be more reliable in some times and places than in others. In fact, one can identify certain times and places in which epistemic authorities have been especially unreliable. For example, some particularists highlight the unreliability of epistemic authorities, and authorities more generally, within the Soviet Union (e.g. Dentith 2014, Chap. 7). In such a context, it seems that the fact that some allegation of conspiracy conflicts with the claims of epistemic authorities is no mark against it.

Three responses to this line of objection are in order. First, even if we can identify certain contexts in which epistemic authorities have been relatively unreliable, this does not impugn the reliability of epistemic authorities in our own contexts. In fact, insofar as identifying unreliable epistemic authorities within certain historical contexts depends on the scholarship of epistemic authorities with which we more closely identify, one cannot deny the reliability of particular epistemic authorities without implicitly affirming the reliability of others.^15^ Thus, one can recognize that some epistemic authorities are unreliable, even while allowing that, in other contexts, there is reason to be skeptical of conspiracy theories. Second, it is not clear from the examples given by particularists that even epistemic authorities within the Soviet Union were so unreliable as to nullify the pessimistic induction in that context. Undoubtedly, such authorities made false claims concerning a range of vitally important issues, but their unreliability can only be assessed by considering the frequency of such claims within the broader body of claims made by the same authorities. Finally, it is important to recognize that the case against *Strong Particularism* does not assume that epistemic authorities are reliable, full-stop. Rather, the assumption is that, at least in many contexts, trusting epistemic authorities as good guides at least with respect to what *not* to believe is the best available strategy with respect to the twin epistemic aims of believing truths and shunning falsehoods.^16^ Consider an analogy. Nutrition science is a notoriously unstable discipline, characterized by persistent controversies and inconsistent recommendations. One might, for this reason, reasonably deny that the claims of nutrition scientists should be taken on trust. However, it does not follow that the fact that a claim is disputed by nutrition scientists is not a strong reason to be skeptical of this claim. Even if the testimony of nutrition scientists is not a sufficient reason to believe a claim, such testimony might be a compelling reason not to believe some other claim with which the former conflicts. This would be the case if, for instance, nutrition science was flawed but nonetheless the best existing social epistemic structure for uncovering truths about nutrition. Similarly, even if one is dubious of relevant epistemic authorities’ claims concerning some conspiracy theory—perhaps because comparable authorities have sometimes been wrong regarding similar claims in the past—one might simultaneously recognize that those authorities are the ones most likely of all parties to arrive at the truth. This may not be a sufficient basis on which to accept the claims of those epistemic authorities, but it is nonetheless sufficient reason for defeasible skepticism of the conspiracy theory in question.

Even if one agrees that the best epistemic policy is to begin with a skeptical attitude toward conspiracy theories, one might argue that there are strong non-epistemic reasons to avoid a generalized suspicion of conspiracy theories. Recalling the particularist concerns discussed in Sect. 3, one might worry that the assignment of low prior probabilities to conspiracy theories is dangerous insofar as it would likely allow some conspiracies to go undetected. But such concerns are misguided. Whatever consequences the attitude of skepticism toward conspiracy theories might have, such consequences are irrelevant to the arguments given above. Moreover, it is far from clear that this attitude of skepticism would have worse consequences than the absence of skepticism. Even if we suppose a skeptical stance toward conspiracy theories would allow some conspiracies to go undetected, this cost is not obviously worse than the costs of assigning relatively high prior probabilities to conspiracy theories. Additionally, because one can investigate conspiracy theories even while maintaining a highly skeptical attitude toward them, it is far from clear that the skeptical attitude would have any negative consequences. Finally, insofar as one remains concerned that the line of argument against *Strong Particularism* would, in some cases, motivate some degree of trust in unreliable epistemic authorities, the proposal here carries with it a straightforward response. If the possibility of unreliable epistemic authorities is the problem, then improving the systems whereby individuals become epistemic authorities is the solution. The importance of this point is most clearly recognizable when we consider concrete cases. In recent years, many conspiracy theories have alleged complex plots, often involving sophisticated technologies. Allegations of widespread electoral fraud in the 2020 US Presidential Election offer an illustrative example. The notion that ordinary individuals should investigate such allegations themselves is highly impracticable—especially because these allegations typically appeal to supposed technological methods of vote switching—and would likely yield little more than a mass of unfounded conspiracy theories and confused refutations of these. A more realistic suggestion is that individuals lacking the relevant expertise should apply what power they possess to shape systems such that positions of epistemic authority are reserved for those possessing the needed expertise.

## Concluding remarks

Particularism is often presented by its explicit defenders as at least approaching consensus status within the philosophy of conspiracy theories. I have sought to challenge this picture. As I have argued here, particularism is best understood not as a single thesis, but rather as a constellation of related theses. Hence, one may be a particularist in some respects and a generalist in others. I have argued that *Strong Particularism*—according to which there are no grounds for a generalized but defeasible skepticism of conspiracy theories—is false. This result does not imply that particularism is, on the whole, mistaken. But it does imply that particularist triumphalism is premature.

## Footnotes

1 Stokes (2018a), who explicitly advocates “defeasible generalism”, is a possible exception.

2 This appears to be the approach taken up by Napolitano and Reuter (2021) who endorse a pejorative definition, while also acknowledging that many individuals regard the term as principally descriptive. I discuss pejorative definitions below.

3 Napolitano and Reuter (2021) provide evidence supporting a pejorative definition according to this metric. However, in part for reasons discussed below, I do not consider this empirical evidence decisive.

4 Thanks to two anonymous referees at *Canadian Journal of Philosophy* for raising this point.

5 One might argue that this is an excessively subjective construal of what it is for there to be evidence for a theory. However, given the plausible assumption that one can only have evidence if there is evidence, it follows that whenever conspirators have evidence of a conspiracy, there is evidence of conspiracy in a more objective sense.

6 The bipartite distinction made here resembles Theodore L. Brown’s distinction between coercive authority and expert authority (2009, pp. 22–23).

7 This description is apparent in accounts from academics (Balkin, 2021), media accounts of the claim (Bailey, 2021; Bump, 2021; Cohen, 2021), rhetoric among political figures (Smith, 2021), and legal rulings (Polantz, 2021).

8 This is not to say that the present definition could be expected to entirely eliminate controversy as to what amounts to a conspiracy theory. Questions may remain, for instance, concerning what degree of conflict with the claims of epistemic authorities is required for some theory to be a conspiracy theory. We are thus left with a sort of threshold problem, familiar from elsewhere in in philosophy, including epistemology (Hetherington, 2006). However, as in other areas of philosophy, the fact that the definition gives rise to a threshold problem does not indicate the falsity of that definition.

9 I do not mean to assert that beliefs in conspiracy theories are typically so caused. This is an empirical claim for which there does not appear to be adequate evidence. Rather, my aim is to establish the coherence of a certain set of attitudes that might otherwise be thought to conflict.

10 Stokes (2018a, b), for instance, emphasizes the moral costs of investigating conspiracy theories. Dentith (2018) argues that these costs can be minimized, while acknowledging that the sheer volume of conspiracy theories presents a practical challenge to investigating them.

11 It is worth emphasizing here that Dentith’s conclusion is not that historical instances of conspiracy alone provide sufficient grounds to rationalize belief in further conspiracy theories. Rather, Dentith’s conclusion is the more modest one that historical instances of conspiracy recommend the assignment of relatively high prior probabilities to further conspiracy theories. The apparent implication of this conclusion, for the rationality of belief in conspiracy theories, is that it takes less evidence specific to a given conspiracy theory to rationalize belief in that conspiracy theory than might otherwise be supposed.

12 See Basham (2011, p. 74) for a similar line of argument.

13 Thanks to an anonymous referee for raising this point.

14 Even if one declines to assume the falsity of any particular subset of conspiracy theories, the fact that large number of conspiracy theories present mutually incompatible explanations of the same events allows one to conclude that most such theories are false. More generally, conspiracy theories tend to conflict with one another insofar as they tend to posit that different groups hold sway over events. Thanks to an anonymous referee at *Canadian Journal of Philosophy* for pressing this point.

15 This brief argument offers a mirror of an argument first offered by Pigden. Pigden argues that, given the commonality of conspiracies within history, every historically literate person must accept some conspiracy theories (Pigden, 2006, p. 157). The present argument, combined with our first pessimistic meta-induction, suggests that every historically literate person has reason to be skeptical of some conspiracy theories. The scope of this argument is limited, however, because it could in principle be the case that epistemic authorities with respect to historical questions are more reliable than epistemic authorities with respect to non-historical questions.

16 This point is not particular to allegations of conspiracy, but echoes the general contention made by some social epistemologists that the best epistemic strategy is to embrace our epistemic dependence on others (Hardwig, 1985).
